# An Examination of Mixed Martial Arts Spectators’ Motives and their Sports Media Consumption in Poland

**DOI:** 10.1515/hukin-2015-0048

**Published:** 2015-07-10

**Authors:** Paweł Zembura, Jolanta Żyśko

**Affiliations:** 1 Department of Organization and History of Physical Culture, Józef Piłsudski University of Physical Education in Warsaw, Poland.; 2 - Warsaw School of Tourism and Hospitality Management, Warsaw, Poland.

**Keywords:** spectator behaviour, combat sports, motivations

## Abstract

The study attempted to analyse the concept of spectators’ motives at mixed martial arts (MMA) events in Poland. In addition, we investigated the relation between motives and sports media consumption. The sample consisted of 273 people attending three similar, regional MMA events. Exploratory factor analysis was used to refine the structure of motives. Confirmatory factor analysis showed a reasonable fit of the obtained model (RMSEA = 0.41). Using ANOVA we found three significant differences in assessment of motives, based on gender. The factor of aesthetics and knowledge was ranked the highest for men and women. Men rated drama and violence, while women perceived socializing and crowd experience, and drama, as the following factors. Path analysis indicated that these motives explained 56% of variance in media consumption for men and 57% for women. The findings showed that the motive of vicarious achievement was the main predictor of media consumption for men, while aesthetics and knowledge were the key predictors for women. The results and ideas for further research are discussed.

## Introduction

Watching sport is one of the most popular leisure activities in Poland, which can be illustrated by more than 10 million domestic applications for tickets at EURO 2012 in Poland and Ukraine (UEFA, 2011). Further, the attractiveness of sporting events transfers well to the media, as sport events have attracted some of the highest rankings in the history of Polish television (Reisner, 2012). Nevertheless, the popularity of particular spectator sports might be considered very sensitive, increasing when a domestic athlete is successful on the international stage and decreasing with his/her setbacks (ARC Rynek i Opinia, 2013). Considering this dynamics, it is remarkable to witness a form experiencing high, steady growth, such as mixed martial arts (ARC Rynek i Opinia, 2013).

Mixed martial arts (MMA) stand for a combat sport, where participants compete using various techniques adapted from disciplines such as boxing, wrestling, Brazilian jiu-jitsu, and Muay Thai. The sport might be described as dynamic, unpredictable, and violent. Due to the latter characteristic, it is also controversial and receives a lot of criticism from public authorities and media (Bottenburg and Heilbron, 2006; Jewell, 2012). The first MMA fight in Poland was hosted in 2002, although the dynamic growth of MMA started in 2006 when KSW, a domestic organization, brought sport celebrities from other disciplines as contestants. At the same time, promotion obtained a slot on a popular television channel, Polsat. Fights involving KSW stars consistently drew more than three million television viewers, with a record of more than five million viewers (Reisner, 2012). Along with increasing success in the media, the sport has increased tremendously at the regional and amateur level. In 2012 about 70 different types of MMA events were organized in Poland. Furthermore, MMA with K-1 was recently ranked as the sixth most popular sport in Poland among avid sport fans (ARC Rynek i Opinia, 2013).

Despite significant growth, many new mixed martial arts organizations fail to remain on the market for a longer period of time. This is mainly due to low attendance at events, as payout from the live gate is a crucial source of their revenue. Therefore, from the sport marketing perspective, it is necessary to recognize factors driving spectators to attend a particular sporting event (Funk, 2008).

This information might be provided by spectator motives, which in this study we considered to be specific psychological features, such as desires and emotions that can influence the behaviour of a sport spectator (Mahony et al., 2002). Extensive studies lead to identifying several core spectator motives (McDonald et al., 2002; Trail and James, 2001). However, it was also acknowledged that some particular motives are related to specific characteristics of an event or sport.

Opposite to team sports, spectators of different martial arts events attracted little interest until the rise of MMA (Kim et al., 2008). The rapid growth of MMA, paired with its violent nature, brought concerns about who is seeking the emotions provided by this sport. Hence, only a few studies aimed to examine the motives of spectators attending events and fans consuming different types of related media have been conducted (Andrew et al., 2009; Brown et al., 2013; Cheever, 2009; Frederick et al., 2012; Kim et al., 2008; Lim et al., 2010; MacIntosh and Crow, 2011).

The essential motives for spectators of MMA were drama, aesthetics, vicarious achievement, and entertainment (Andrew et al., 2009; Kim et al., 2008, 2009). Due to the uniqueness of the sport, a few less popular dimensions of motives were added to the scales, such as violence, crowd experience, adoration, national pride, economic factor, and knowledge (Andrew et al., 2009; Kim et al., 2008, 2009). Although a few of them were found unsuitable, others showed to vary depending on the country or demographics. For example, males rated the motive of violence higher than females (Andrew et al., 2009; Kim et al., 2008). In the cross-national study, Kim et al. (2009) found that audiences in the United States were more motivated than in South Korea taking into account aesthetics, while less motivated by national pride and vicarious achievement. Referring to cultural differences between spectators’ motives in MMA, previous studies concluded that consecutive research should be undertaken in other areas where the sport has experienced growth (Kim et al., 2009). Furthermore, motives have explained media and merchandise consumption as well as willingness of future attendance, although their individual contribution to the studies was inconsistent (Andrew et al., 2009; Kim et al., 2008, 2009).

The objectives of this paper might be divided into two groups. First, the aim was to unveil the essential dimensions of the combat-sport specific construct of spectators’ motives. Thus, factor analysis was performed to refine the underlying structure of motivational factors. Second, we studied motivational patterns regarding MMA consumption in Poland. We investigated which motives were prevalent for attendance. Previous studies had shown distinctions in these preferences based on spectators’ gender. Consequently, we tested whether the motives differed depending on the gender of a spectator. Finally, media consumption plays a crucial role in the development of the sport. Therefore, we explored whether the motives to attend an MMA event were a predictor of the sports media consumption.

## Material and Methods

### Study design

The design of the study can be separated into three stages. First, the questionnaire, which was developed and used in the Andrew et al.’s (2009) study, was translated into Polish. Due to this process and cultural adaptation of the instrument, it was decided to check its internal consistency before the full-scale research process. For this reason, we performed a pilot study carried out on the sample of MMA fans voluntarily recruited from the biggest thematic website on Polish MMA. Participants attended at least one MMA event. This pre-test was conducted online, using the SurveyGizmo website. Based on the results of 70 fully filled questionnaires, it turned out that four subscales lacked internal consistency (Cronbach’s alpha <0.7). They were reformulated and underwent additional cultural adaptation. The refined version of the questionnaire was used in further research. In the full project, the sample was limited to the people attending MMA events. All the forthcoming information refers to that phase.

### Sample

The sample consisted mainly of young males (Table 1). The events showed rather regional interest.

### Measures

The questionnaire consisted of 37 items, divided into 4 groups:

1. Demographic, economic, and geographic information (6 items).
2. Motives (27 items). The scale of motives was adapted from the Andrew et al.’s (2009) study, which arose due to a number of reasons. The instrument was specifically designed to capture the specifics of MMA spectatorship. It showed acceptable consistency during research at professional MMA events. Further, we intended to use the same instrument, as it would eventually allow us to compare the results cross-nationally. Respondents had to rate their opinions on the 7-point Likert scale, ranging from 1 – “strongly disagree” with a presented statement, to 7 – “strongly agree”. The scale of motives consisted of the following initial subscales: crowd experience, escape, drama, adoration, socializing, vicarious achievement, aesthetics, knowledge, and violence (Andrew et al., 2009). In the Andrew et al.’s (2009) study, the scale showed acceptable reliability with a Cronbach’s alpha of subscales ranging between 0.76 and 0.88. Although most of these motivational dimensions are well established in the literature, some might be considered specific to MMA, and thus will be shortly described. Adoration refers to the hero status of an athlete that might influence consumers’ behaviour (Bush et al., 2004; Dix et al., 2010; Kim et al., 2008). Violence is supposed to be a factor that increases attractiveness and entertainment value of a sport contest, although it has been rarely tested in empirical research (Jewell, 2012; Russell, 2008). As mixed martial arts are frequently considered and promoted as “real fights” and the amount of sanctioned physical force that occurs during the contest is high, it was believed that violence would attract people seeking such amusement, and for that reason subsequent studies included this motive (Andrew et al., 2009; Kim et al., 2009). Crowd experience stands for belonging to part of a larger group and “feeling their emotions”, which was distinguished as a dimension of sport event entertainment value (Andrew et al., 2009). The motive of knowledge refers to attending an event in order to learn about particular qualities of a sport, such as technique and strategy (Andrew et al., 2009).
3. Sport consumption (two items). To describe sports media consumption, we used two factors established by Fink et al. (2002) and adapted to the MMA context by Kim et al. (2008). These were: “I read about MMA news over the Internet”, “I watch MMA events on television”. MMA, as a sport of the “new era”, is broadly present on the Internet, particularly in social media (Broughton, 2012). In addition, both domestic and international MMA events are shown on Polish television. The third factor used in the Kim et al.’s (2008) study, which referred to watching MMA reality shows on television, was not included. Although such programmes have most recently been produced in Poland, they are not popular, being accessible just on niche channels and on the Internet.
4. Sport experience (two items). Spectators were asked how many MMA events they had previously attended. Additionally, we inquired how they had first learned about MMA.

### Procedures

The data was gathered during three similar events, representative of the most popular regional shows in Poland. The events were small (500–1200 spectators), professional, and focused on evenly matched contests. They were placed in the towns between 80,000–200,000 inhabitants in South-East and Central Poland. Promotions were local and originated in the cities of the events. We received permission from the organizers to distribute questionnaires. Participation was voluntary. The questionnaires were distributed prior to the event, as people were allowed into the sporting arena. After taking their seat, they were asked to fill out the questionnaire and leave it on their seat afterwards. VIP seats were excluded from the study. The first research was conducted in March 2013 and the last one in November 2013. Out of 335 distributed surveys, 318 were returned (95% return rate), and 273 were usable.

### Statistical analysis

#### Factor analysis

Exploratory factor analysis (EFA) was performed to identify the underlying relationships between observed variables and to examine factorial validity of the latent variables proposed in the instrument (Conway and Huffcutt, 2003). We considered it appropriate for several reasons to use EFA in order to provide a basis for specifying a concept of motives (Matsunaga, 2010). The instrument had undergone translation and cultural adaptation. Its original version had not been extensively tested. In addition, it is specific to the combat sport context, and for that reason includes some dimensions of motives that have limited empirical background, such as violence, adoration, and crowd experience. Moreover, in the previous studies, strong correlations were found among some variables, such as crowd experience and socializing (Andrew et al., 2009; Kim et al., 2008).

Prior to EFA, we tested assumptions for normality by assessing skewness and kurtosis for each item. For EFA we used the maximum likelihood extraction method with varimax rotation. Suitability of the data was assessed by KMO statistic and the Bartlett’s Test of Sphericity. Factors were distinguished based on analysis of the theoretical background, scree plot, and eigenvalues (Tabachnick and Fidell, 2007). The structure of items was refined by using a pattern matrix and excluding observed variables, which were strongly inter-loading (with a difference below 0.2 on main and other items), not loading on any factor, or had low loadings, below 0.32 (Tabachnick and Fidell, 2007).

#### Confirmatory factor analysis

Confirmatory factor analysis (CFA) in AMOS 20 was conducted to examine the obtained structure of motives. For measuring the model fit, we analysed the normed chi-square (CMIN/DF), root mean square error of approximation (RMSEA), comparative fit index (CFI), and standardized root mean square residual (SRMR). As for CMIN/DF, the acceptable threshold level was below 3 (Kline, 2005). Values of RMSEA of less than 0.08 imply an acceptable model fit, while values of less than 0.05 imply a good fit (Kline, 2005). The value for CFI was expected to be over 0.9 (Browne and Cudeck, 1989). Values for the SRMR lower than 0.08 are considered acceptable (Hu and Bentler, 1999). Items convergent validity was assessed by average variance explained (AVE) factor loading (AVE over 0.5). Inter-item correlations were analysed to evaluate discriminant validity and were expected not to exceed 0.8 (Brown, 2006). Additionally, we expected maximum shared variance (MSV) to be lower than average variance explained (AVE) and average shared variance (ASV) to be lower than AVE, based on guidelines provided by Hair et al. (2010). Internal reliability was determined by Cronbach’s alpha and component reliability (CR) using a cut-off value of 0.7 (Hair et al., 2010).

#### Ranking of the motives

To estimate factor scores for motives and media consumption we computed composites using the regression method in AMOS.

#### Differences between gender

Using these values we compared mean scores for motives between male and female spectators utilizing one way analysis of variance (ANOVA). Effect size was estimated using Cohen’s d, where values greater than 0.20, 0.50, 0.80 suggest small, medium, and high effect size, respectively (Cohen, 1988). Previously, we tested assumptions for ANOVA: homoscedasticity, multicollinearity, and distribution of the items.

#### Predictors of sports media consumption

Two separate path analyses were conducted for each gender to find predictors of media consumption. First, we checked assumptions of path analysis using curve estimation in SPSS. Non-significant items (p < 0.05) were excluded to obtain parsimonious models.

Except where stated otherwise, statistical analysis was conducted in SPSS 20.

## Results

### 

#### Exploratory factor analysis

Factor analysis using the Maximum Likelihood Method with promax rotation produced the six-factor solution (Table 2). Two of the distinguished common factors consisted of more than one initial concept. It paired aesthetics and knowledge in one factor and socializing plus crowd experience in another. These relationships between constructs were expected and the retained factors were theoretically consistent and interpretable. Thus, it was decided to keep the labels describing the variables during the whole analysis. Other distinguished motives were vicarious achievement, violence, escape, and drama. Single items belonging to escape, socializing, and knowledge, as well as the whole subscale of adoration, were dropped as they had low loading or were strongly loading between items. After the analysis, 21 observed variables were retained in the model. Six extracted latent variables accounted for 59% of the total variance. The KMO measure was 0.837 and the Barlett’s Test of Sphericity reached the level of significance of d < 0.001, confirming the appropriateness of factor analysis in the sample. Among the accepted items, assumptions for normality assessed by skewness (ranging between −1.7 and −0.3) and kurtosis (values between −0.9 and 2.8) were met.

#### Confirmatory factor analysis

In order to address model fit, we were compelled to covary error terms that were a part of a single factor.

Model fit was acceptable with CMIN/DF=1.924, CFI=0.910, RMSEA=0.41, and SRMR= 0.0577. The model met the threshold for reliability (CR > 0.7) and discriminant validity: MSV < AVE, ASV < AVE (Hair et al., 2010) (Table 3). Cronbach’s alpha of the factors were acceptable, ranging from 0.75 for drama to 0.85 for socializing and crowd experience, violence, and escape. Cronbach’s alpha value for media consumption was 0.7, which met the border line.

#### Ranking of the motives

The means of the obtained composite variables indicated that a factor of aesthetics and knowledge (5.46 ± 0.89 for men, 5.43 ± 0.9 for women) was the highest ranked motive among MMA spectators in Poland. The following motive varied depending on gender, as it was drama for men (5.35 ± 0.98), and socializing and crowd experience for women (5.35 ± 1.2) (Table 4). Violence was ranked as the third most important factor for men (5.26 ± 1.47), and the least important for women (4.16 ± 1.72).

#### Differences between gender

Testing assumptions for ANOVA, we found no multicollinearity issues and no problems with normality (between −1.2 and −0.3 for skewness, between 0.3 and 1.9 for kurtosis) of the items, although the Levene’s Test for Equality of Variances showed that with regard to violence, variability between the scores for women and men was significantly different at p < 0.05. For this reason we used Welch’s ANOVA.

Gender had a significant effect on three motives at p < 0.05 (Table 4). Cohen’s effect size (p < 0.001, d = 0.69) suggested moderate significance for violence and small practical significance for socializing and crowd experience (p < 0.01, d = 0.35) and vicarious achievement (p < 0.05, d = 0.26).

#### Predictors of sports media consumption

The model for men predicted 56% of variance in sports media consumption. Four motives contributed to explaining media consumption at the level of significance of p < 0.05 (Table 5). The motive of vicarious achievement (β = 0.388) was found to be the strongest predictor, followed by aesthetics and knowledge (β = 0.323). The model for female spectators accounted for 57% of variance. Analysis revealed that three motives contributed to explaining variance in media consumption. Aesthetics and knowledge were the strongest predictors of media consumption among women (β = 0.696).

## Discussion

The main purpose of the study was to analyse the motives of MMA spectators and their relation with sports media consumption in Poland. The findings provide a better understanding of MMA spectatorship from a regional perspective and give an opportunity to compare the results with other countries. Although the popularity of MMA in Poland has grown far from the global mainstream, the core of spectators’ motives remains relatively consistent with other nations. Nevertheless, based on spectators’ gender, pivotal differences in motives and their relation to media consumption were acknowledged.

### Motives of attendance

Results from this study show that the most important motives for MMA spectators in Poland are aesthetics and knowledge, drama, as well as crowd experience and socializing. The aesthetic qualities of sport contests along with drama were consistently found essential in studies regarding attendees’ motives at MMA events (Andrew et al., 2009; Kim et al., 2008, 2009; MacIntosh and Crow, 2011), except for the Korean sample in the Kim et al.’s (2009) study.

Dominance of the aesthetics and knowledge motive in Poland is a key finding. As it refers to the appreciation of fighters’ skills and willingness to learn about the sport, it should be clearly stated that MMA spectators declare seeking high quality sport performance to be foremost. That is, they are able to find skilfulness and sport mastery in a violent sport such as MMA.

The result applies to the long-term discussion on whether aggression and violence in sport might go together with appreciation of aesthetic values for fans.

Russell (2008) stated that aesthetic values and violence coexist in some sports, as even the fanciest and most skilful performance in boxing would not bring attention to the sport if the fighters were not allowed to hit each other. Nevertheless, the magnitude of aesthetics among MMA spectators is in contrast to a more general examination of motivational patterns among sports fans of nonaggressive and aggressive sports (Wann et al., 2008; Wann and Wilson, 1999). In one of their studies, Wann et al. (2008) concluded that the enjoyment of grace and quality of competition were “turned off” by aggression and violence in sport.

Another highly ranked motive was drama. Due to the complexity and repertoire of the allowed techniques, MMA is highly unpredictable and for that reason might attract people looking for exciting, intense contests. Andrew et al. (2009) mentioned that drama might be a factor that divides MMA from boxing, which at a local level frequently consists of predictable fights, where the disproportion between the skills and records of competing fighters is evident. In contrast, at MMA events where the data for the present study was collected, fighters were evenly matched, having comparable fighting records and professional experience.

### Differences between motives based on gender

In the study we found that the order and assessment of particular motivations of attendance varied depending on gender, which confirms that other marketing strategies have to be applied to attract male and female spectators. While some motivational differences associated with gender were met in previous studies in the MMA context (Andrew et al., 2009; Kim et al., 2008), in Poland these were explicit and referred to half of the factors.

The most decisive difference was acknowledged with regard to violence, which was rated as the third most important motive for men and the least important for women. In their work, Kim et al. (2008) stated that, contrary to critics of MMA, violence was not among the most important motives for spectators. Yet, the medium effect size of gender in our research as well as its high place in the hierarchy of motives for males is a clear sign that this factor should be analysed according to spectator’s gender. Additionally, given that this particular motive is not socially desirable and respondents might be biased when declaring their interest, it should be articulated that at some MMA events violence might play a pivotal role in attracting male spectators.

Another difference was observed with respect to socializing and crowd experience, which were more important for women. As the sport of MMA primarily attracts young males (Cheever, 2009), women attending the shows might be frequently interested in social interactions at the event rather than in the sport itself (Kim et al., 2008). Opposite to preferences of spectators in the United States, in our study we also found that women rated vicarious achievement higher than men (Andrew et al., 2009; Kim et al., 2008).

### Relations between motives and sports media consumption

The spectatorship motives explained a large proportion of variance in sports media consumption (56% for men, 57% for women), which is a similar result to corresponding studies, where motives accounted for between 41% and 56% of media consumption (Andrew et al., 2009; Kim et al., 2008, 2009).

The motives of aesthetics and knowledge as well as violence were predictors of sports media consumption shared between men and women. Likewise, aesthetics was the strongest positive predictor in the Andrew et al.’s (2009) study, and it seems understandable that those among the spectators who appreciate excellence in MMA would be the most interested in media consumption (Andrew et al., 2009; Kim et al., 2008). Comparison of the result with prior research exposes that it is interest in particular sports that mostly distinguishes committed sports media consumers among spectators (Kim 2008), and the motives of aesthetics and knowledge were continually theorized as preceding fans’ attachment to a sport (Trail et al., 2003; Woo et al., 2009).

The motive of violence was a significant and positive predictor of media consumption. The relation was observed in male spectators in the Andrew et al.’s (2009) study, which once more shows that this controversial aspect of MMA should not be ignored when thinking about factors attracting people to the mediated sport. The result confirms a more general finding that some sports media consumers enjoy watching violence and aggression (Jewell, 2012; Russell, 2008).

The vicarious achievement motive was the most important predictor of sports media consumption for male spectators. Even though such relation was found in the Kim et al.’s (2009) study regarding MMA in North America, it was not critical as it was in our study. This outcome suggests that male spectators who look to fulfil the need of achievement by associating themselves with successful athletes would be more engaged in sports media. Interestingly, MMA-related media consumption in Poland is somehow limited due to the small number of events in larger media outlets. Since MMA fans are enthusiasts of social media, this is probably how they keep relationships with fighters (Broughton, 2012).

The negative relation between the socializing and crowd experience motive and media consumption might refer to lower interest in the sport among people primarily attracted by this motive. Additionally, there are not many opportunities to link socializing with consumption of the mediated sports content in Poland. Fans of MMA who are willing to watch regional events usually need to seek replays on the Internet or small, dedicated television channels.

## Conclusions

Spectators of MMA in Poland were foremost motivated by aesthetics and knowledge. The comparison of mean scores for motives and their hierarchy suggest that for men, features of MMA events related to the sporting aspect of the show and fighters’ performance were more attractive.

Another finding is that people motivated by aesthetics and knowledge or violence, which are sport-specific, are more willing to consume MMA media content, while those seeking event-related features would be less interested in such consumption. However, in relation to similar studies, the predominance of vicarious achievement as a positive predictor of sports media consumption in the male sample was extraordinary (Andrew et al., 2009; Kim et al., 2008, 2009).

The results of this study may not be representative for Polish MMA as a whole, as the sample consisted only of individuals attending small, regional shows, emphasizing the sporting aspect of a competition. Spectators’ motives might differ among those attending greater events with a broader set of attractions (concerts, fights between sport celebrities, etc.). Another step into exploring the characteristics of MMA spectators should be to measure their attachment towards sports entities involved in an event. As found in several studies, MMA fans have some specific features; for example, they are young, use a lot of social media, and have an extraordinary perception of violence in sport (Andrew et al., 2009; Broughton, 2012; Cheever, 2009). Their comparison with spectators of traditional combat sports such as boxing may reveal other qualities of this group of consumers.

## Figures and Tables

**Table 1:** Basic characteristics of the study sample

Gender	Male	68%
	Female	32%

Age	18–24	43%
	25–29	26%
	30–39	27%
	40–49	3%
	50–59	1%

Education	university	53%
	secondary	37%
	vocational	6%
	elementary	2%
	no education	1%

Place of living (number of inhabitants in thousands)	town over 500	15%
	between 200–500	6%
	between 100–200	14%
	between 20–100	27%
	town of 20 or less	10%
	countryside	28%

Do they live in the voivodship of this event	yes	85%
	no	15%

Median income per household member in zł (in quintiles)	1651 zł and more	48%
	1201–1650	20%
	1200–901	13%
	900–641	9%
	640 or less	10%

How many MMA events have they attend before this event	0	26%
	1	25%
	2 or 3	25%
	4 or more	24%

How have they learned about MMA events	from TV	25%
	from the Internet	21%
	from friends/relatives	43%
	from advertisements	11%

**Table 2:** Results of exploratory factor analysis

item:	factor					
	1	2	3	4	5	6
...the crowd energy I feel at the event gets me pumped up	0.82					
...there is something special about being in a crowd.	0.80					
...I feed off the excitement of the crowd	0.74					
...of the chance to socialize with other fans.	0.69					
...of the opportunity to interact with other fans.	0.51					
...MMA is a form of art.		0.80				
...I can learn about the technical aspects of MMA.		0.74				
...of the graceful agility displayed by MMA fighters.		0.68				
...watching a well-executed athletic performance is something I enjoy.		0.67				
...I increase my understanding of MMA strategy by watching an MMA event.		0.51				
...I enjoy the brutality of MMA.			0.97			
...I like an MMA event more when the fights get bloody.			0.81			
...I like MMA because it has more violence than other sports.			0.65			
... I feel a personal sense of achievement when my favourite fighter does well.				0.92		
...I feel like I have won when my favourite fighter wins.				0.63		
...I feel proud when my favourite fighter does well.				0.51		
...I enjoy the drama of close fights.					0.73	
...I enjoy a close fight more than a one-sided fight					0.73	
...I enjoy fights where the outcome is not decided until the very end.					0.69	
...it provides a distraction from my every day activities.						0.88
...it provides an escape from my day-to-day routine.						0.79
Cumulative % of variance	26.38	37.76	44.68	51.8	55.99	59.11
Eigenvalues	6.53	2.42	1.82	1.75	1.14	1.01

Maximum Likelihood Method with promax rotation

Factor loadings below 0.35 were suppressed

**Table 3:** Scale Reliability and Intercorrelation Matrix between motives

	CR	CA	AVE	MSV	escape	soc&ce	vic.ach	ae&kn	vio	drama
escape	0.857	0.853	0.750	0.340	0.866					
soc&ce	0.845	0.848	0.524	0.340	0.583	0.724				
vic.ach.	0.755	0.789	0.510	0.376	0.281	0.564	0.714			
ae&kn	0.835	0.819	0.502	0.376	0.285	0.469	0.613	0.709		
vio	0.858	0.847	0.673	0.146	0.135	0.189	0.161	0.382	0.820	
drama	0.758	0.754	0.513	0.339	0.413	0.389	0.301	0.582	0.221	0.716

CR - construct reliability; CA - Cronbach’s alpha; AVE - average variance explained; MSV - maximum shared variance; soc&ce - socializing and crowd experience; vic.ach - vicarious achievement; ae&kn - aesthetics and knowledge; vio - violence

**Table 4:** Descriptive statistics of results and ANOVA

	together		male		female		ANOVA		
motive	mean	SD	mean	SD	mean	SD	F	p	Cohen’s d
aesthetics & knowledge	5.45	0.89	5.46	0.89	5.43	0.90	0.08	0.783	0.04
drama	5.30	1.02	5.35	0.98	5.18	1.10	1.56	0.213	0.16
socializing & crowd experience	5.06	1.17	4.94	1.14	5.35	1.20	7.38	0.007	0.35
violence	4.91	1.63	5.26	1.47	4.16	1.72	29.54	<0.001	0.69
escape	4.78	1.20	4.70	1.15	4.96	1.27	2.94	0.088	0.22
vicarious achievement	4.54	0.92	4.46	0.93	4.70	0.90	4.01	0.04	0.26

**Table 5:** Significant paths between spectators’ motives and media consumption and the proportion of variance explained

		coefficient (b)	S.E.	st. coefficient (β)	C.R.	p	R^2^

women							0.567
	aesthetics & knowledge	0.983	0.133	0.696	7.389	<0.001	
	violence	0.187	0.059	0.253	3.156	0.002	
	socializing & crowd experience	−0.191	0.094	−0.180	−2.043	0.041	
		coefficient (b)	S.E.	st. coefficient (β)	C.R.	p	R^2^
men							0.562
	vicarious achievement	0.485	0.098	0.388	4.964	<0.001	
	aesthetics & knowledge	0.418	0.117	0.323	3.581	<0.001	
	drama	0.239	0.079	0.203	3.034	0.002	
	violence	0.129	0.044	0.164	2.970	0.003	
	socializing & crowd experience	−0.188	0.067	−0.185	−2.828	0.005	
